# Liposomal and Lipid-Based Drug Delivery Systems: Bridging Gut Microbiota and Pediatric Disorder Treatments

**DOI:** 10.3390/pharmaceutics17060707

**Published:** 2025-05-28

**Authors:** Raluca Ioana Teleanu, Elena-Theodora Moldoveanu, Adelina-Gabriela Niculescu, Elena Predescu, Eugenia Roza, Iulia Florentina Tincu, Alexandru Mihai Grumezescu, Daniel Mihai Teleanu

**Affiliations:** 1 Department of Neuroscience, “Carol Davila” University of Medicine and Pharmacy, 020021 Bucharest, Romania; raluca.teleanu@umfcd.ro (R.I.T.); daniel.teleanu@umfcd.ro (D.M.T.); 2 Pediatric Neurology Department, “Dr. Victor Gomoiu” Clinical Children’s Hospital, 022102 Bucharest, Romania; 3 Department of Science and Engineering of Oxide Materials and Nanomaterials, Politehnica University of Bucharest, 011061 Bucharest, Romania; elena.moldoveanu99@upb.ro (E.-T.M.); adelina.niculescu@upb.ro (A.-G.N.); agrumezescu@upb.ro (A.M.G.); 4 Research Institute of the University of Bucharest—ICUB, University of Bucharest, 050657 Bucharest, Romania; 5 Department of Neuroscience, Psychiatry and Pediatric Psychiatry, “Iuliu Hatieganu” University of Medicine and Pharmacy, 400658 Cluj-Napoca, Romania; predescu.elena@umfcluj.ro; 6 Clinical Emergency Hospital for Children Cluj-Napoca, Clinic of Pediatric Psychiatry, 400394 Cluj-Napoca, Romania; 7 Department of Paediatrics, “Carol Davila” University of Medicine and Pharmacy, 020021 Bucharest, Romania; iulia.tincu@umfcd.ro; 8 Pediatric Gastroenterology Department, “Dr. Victor Gomoiu” Clinical Children Hospital, 030167 Bucharest, Romania

**Keywords:** gut microbiota, children neurodevelopmental disorders, liposomal drug delivery systems, solid lipid nanoparticles, probiotics, prebiotics

## Abstract

The intestine is an important segment of the gastrointestinal tract, which is involved in complex processes that maintain the body’s normal homeostasis. It hosts a vast, diverse, and dynamic microbial community called the gut microbiota, which develops from birth. It has been observed that the gut microbiota is involved in essential physiological processes, including the development of the central nervous system via the gut microbiota–brain axis. An alteration of the gut microbiota can lead to serious health problems, including defective neurodevelopment. Thus, this paper aims to highlight the most recent advances in studies that focus on the link between the gut microbiota and the evolution of neurodevelopmental diseases in children. Currently, studies show that the use of drugs that stimulate and restore the gut microbiota (e.g., probiotics and prebiotics) have the potential to alleviate some of the symptoms associated with conditions such as Autism Spectrum Disorder, Attention Deficit Hyperactivity Disorder, Tic Disorder, Tourette Syndrome, epilepsy, and Down Syndrome. In addition, due to the challenges associated with drug administration in children, as well as the widespread shortage of medications intended for pediatric use, researchers are working on the development of new delivery systems. Liposome-based systems or solid lipid nanoparticles have been safely used for drug delivery in various pediatric conditions, which may also indicate their potential for use in the administration of microbiota-modulating therapies.

## 1. Introduction

The gastrointestinal (GI) tract may act as a barrier between the host and external environmental factors, such as food, microorganisms, and antigens. The microbial density in the colon is expected to be between 10^11^ and 10^12^ cells/mL [1,2]. The gut microbiota (GM) hosts about 100 trillion microbial cells, helping to encode more than 3 million genes, and is composed of a wide variety of bacteria, viruses, fungi, archaea, yeasts, and parasites, which together form a biomass of about 1 kg [1,3]. The human gut contains six dominant bacterial phyla—*Firmicutes*, *Bacteroidetes*, *Actinobacteria*, *Proteobacteria*, *Fusobacteria*, and *Verrucomicrobia*—with *Firmicutes* and *Bacteroidetes* (90% of the microbial population) [1,3,4,5,6,7].

The GM represents an essential part of the GI tract that is unique to each individual. It provides a symbiotic relationship between microorganisms that affects numerous physiological functions, including host nutrient metabolism, inflammation, the maintenance of the structural integrity of the gut mucosal barrier, immunity, neurological conditions, and children’s physical and mental development [1,2,8,9,10]. The metabolic pathways of the human body are influenced by the presence of a large number of enzymes in which the GM is involved. Thus, it can lead to the production of bioactive peptides (e.g., branched chain acids, short-chain fatty acids, neurotransmitters, and gut hormones), the synthesis of vitamins (e.g., biotin, folate, pantothenic acid, and vitamin K), and the conversion of bile acids [11,12]. Different factors, such as host-related factors (e.g., age, genetics, general health, intestine pH, peristalsis and transit time, mucus secretions, tissue oxidation–reduction potentials, and mucous immunoglobulin), external factors (e.g., dietary factors and medication), as well as microbial factors (e.g., nutrient availability, bacterial cooperation or antagonism, and bacterial adhesion), can influence the GM balance and can produce a state of dysbiosis (the imbalance of the normal gut microbiota) [9,13,14].

**Figure 1:**
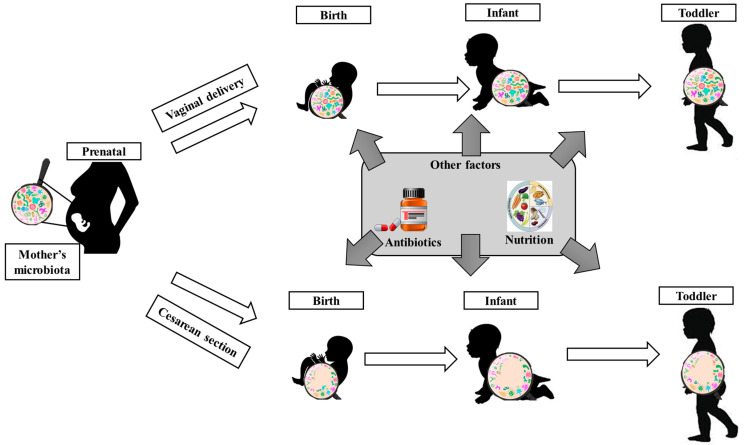
Factors influencing children’s microbiota. Realized based on information from [14,19].

The GM is believed to develop from birth (Figure 1) and undergo a complex and dynamic colonization process during the maturation process [2,5]. The main factor affecting the GM is the mode of birth. It has been observed that newborns born vaginally present a similar microbiota to the mother’s vaginal microflora, which is rich in species such as *Lactobacillus*, *Prevotella,* and *Sneathia*. In contrast, babies born by cesarean sections present species (e.g., *Staphylococcus*, *Corynebacterium*, and *Propionibacterium*) and *Streptococcus* that are predominantly present on the mother’s skin or from the hospital environment. In addition, these infants have been observed to show a delay in colonization with *Bacteroides* species and lower levels of *Bifidobacterium*, which are of increased importance in metabolic health and immune system development [2,5,15]. Breastfeeding can considerably influence the GM, as it supports colonization with species such as *Bifidobacterium*, which develop due to oligosaccharides from breast milk and lead to immune system maturation. In contrast, formula-fed infants show a more diverse microbiota but with increased levels of harmful bacteria [16]. In the first weeks and months of life, the presence of *Clostridium* species consumes oxygen and creates conditions for the development of *Bacteroides* and *Bifidobacterium* species [5]. Thus, changes constantly occur that lead to immune regulation and metabolic development [17]. After 12 months of age, the GM of infants shows *Akkermansia muciniphila*, *Bacteroides*, *Veillonella*, and *Clostridium* species, which continue to stabilize and diversify [1]. Around the age of 2–4 years, the children’s microbiota reaches maturity, and species such as *Firmicutes*, *Bacteroidetes*, and *Actinobacteria* predominate, with families such as *Lachnospiraceae*, *Ruminococcaceae*, *Bacteroidaceae*, and *Bifidobacteriaceae* [1,18]. However, some studies have shown that the differences between the microbiota of babies born vaginally and those born by cesarean sections become less pronounced, indicating the ability of the microbiota to adapt [15].

It was observed that dysbiosis was associated with different disorders in children, such as metabolic disorders (obesity or malnutrition), neurodevelopmental disorders, autism, Attention Deficit Hyperactivity Disorder, and immune system conditions such as asthma and allergies [20,21]. Different drugs and other strategies are used to treat dysbiosis. Changing the diet together with taking prebiotics and probiotics is an essential step in rebalancing the GM. However, treatments for pediatric patients are challenging to obtain, and options are currently limited [22]. The pharmaceutical products used are generally intended for adults and are not indicated for children, as there are uncertainties about the safety and efficacy of the product. Also, the off-label use of pharmaceutical formulations is even more difficult for infants and children up to two years of age [23]. In this respect, studies should focus on producing new medicines specifically designed to treat children.

Lipid-based nanoparticles are ideal candidates for delivering hydrophobic or poorly permeable drugs, presenting advantages such as low clearances and the possibility of increasing the half-life of drugs in plasma [24]. Pediatric medications must be specifically tailored to a child’s age, size, physiologic state, and therapeutic needs [25,26]. In this regard, these nanoparticles have been demonstrated as safe for pediatric drug delivery for various conditions such as cancer, hypertension, human immunodeficiency virus (HIV), and systemic fungal infections [24].

Considering that the GM is involved in a multitude of physiological processes, its damage can cause major disturbances in the normal development of children. Thus, this work aims to highlight the importance of the GM, how it influences neurodevelopment in children, and how the administration of formulations with GM modulatory potentials ameliorates the symptoms of neurodevelopmental diseases. It is also intended to highlight the use of liposomes to manufacture formulations suitable for pediatric administration and the advantages they may have in this regard. Moreover, this work points out the need for further research to acknowledge their importance and role in developing new therapy strategies for neurodevelopmental disorders in children and their need in clinical practice to improve the patients’ quality of life. For this purpose, English language research articles, reviews, and clinical trials were selected using information from scientific databases, such as PubMed, Web of Science, ScienceDirect, MDPI, Scopus, Frontiers, SpringerLink, and Wiley Online Library, using a variety of combinations among the following keywords: “gut microbiota”, “gut-related pediatric disorders”, “gut microbiota in neurodevelopmental disorders”, “gut microbiota in ADHD”, “gut microbiota in ASD”, “gut microbiota in TD” “gut microbiota in DS”, “lipid-based drug delivery systems”, “liposomes”, and “solid lipid nanoparticles”.

## 2. Gut-Related Pediatric Disorders

### 2.1. Gut Microbiota in Children’s Metabolism and Growth

Early microbial colonization represents an important path for metabolic and immune systems, defining the long-term growth trajectories in children’s development [27]. Nutrition, genetics, and the GM composition influence children’s growth trajectories (e.g., normal, delayed, or excessive). If the gut is abnormally colonized or does not reach the necessary maturity, these aspects might affect their growth, metabolism, correct cognitive development, and immune system dysregulation [28]. The gut microbiota remains developmentally immature in malnourished children, even after nutritional interventions. Yet, this immaturity affects the absorption of nutrients and continues the vicious cycle of undernutrition [20,27].

Additionally, several gut microbiota-related metabolites are involved in regulating growth. Yet, the GM represents an essential key factor involved in immune system activity, the regulation of the metabolism, and the entire body’s health. It can maintain the interface between the intake and the host’s physiological functions. In this respect, studies showed that carbohydrates, fats, and proteins influence the microbial composition and function of the gut [29,30]. Carbohydrates, such as complex fibers and resistant starches, go through a process called fermentation, which is realized in the colon, where short-chain fatty acids (SCFAs) (e.g., acetate, propionate, and butyrate) are produced [30]. These SCFAs are associated with important functions that maintain colon health, appetite regulation, and the systemic metabolism. Moreover, these compounds work through G protein-coupled receptors by inhibiting histone deacetylase so that they may influence the intestinal barrier integrity, insulin secretion, inflammation, and gene expression. Thus, butyrate serves as an energy source for colonocytes and has anti-inflammatory properties, while acetate and propionate support gluconeogenesis and satiety, lipogenesis, and appetite signaling [29,30].

However, studies have shown that changes in the gut microbial composition may favor the development of obesity, as well as the metabolism and fat storage which are influenced. Childhood obesity has become a critical global health issue. It is associated with a reduced diversity of the GM and with a high abundance of *Firmicutes* and *Prevotella* species, while *Bacteroidetes*, *Bifidobacteria*, and *Akkermansia muciniphila* levels are low [31,32]. In dysbiosis, SCFAs can be altered by GM dysfunction, which impairs gut health and contributes to obesity-related inflammation and metabolic imbalances [32,33]. Moreover, it was observed that dietary behaviors can influence the GM. So, a high-carbohydrate and low-fat diet has been associated with increased levels of beneficial bacteria (e.g., *Bacteroides* and *Bifidobacteria*). These bacteria decrease the risk of metabolic disorders, while carbohydrate restriction can create a dysregulation in the abundance of butyrate-producing bacteria (e.g., *Roseburia* and *Eubacterium rectale*), leading to a lower SCFA production. Thus, a lower SCFA production conduces an increased risk of potential inflammation [29]. Unlike carbohydrates, protein fermentation in the colon is responsible for both beneficial and harmful compounds. Being in excess, proteins reach the colon, where the microbiota helps in their degradation, obtaining energy and secondary compounds (e.g., phenols, ammonia, indoles, and branched-chain fatty acids) [29,34].

The GM can be influenced by protein through amino acid compositions. Branched-chain amino acids (BCAAs) (e.g., leucine, isoleucine, and valine) promote *Akkermansia* and *Bifidobacterium* growth, bacteria that are involved in gut development and metabolic regulation. Additionally, amino acids, such as tryptophan, can be metabolized in compounds such as serotonin, which play a key role in gut–brain communication [29,35]. Meanwhile, amino acids like lysine and glutamate affect hormone levels and the energy balance [29]. However, the microbiota is involved not only in the breakdown of amino acids but also in their synthesis. Gut bacteria use nitrogen obtained from dietary or internal sources to produce essential amino acids, like lysine or valine, supporting host metabolism and antioxidant defense systems such as glutathione (GSH) [30]. However, the GM also has effects on bile acids and potentiates metabolism with their help, as microbial bile salt hydrolase (BSH) leads to secondary bile acids that are intended to activate receptors such as FXR and TGR5, which implies an improved lipid and glucose metabolism, producing energy [27,36]. In addition, the GM may also lead to the production of lipopolysaccharides (LPSs) derived from Gram-negative species (e.g., *Proteobacteria* and *Bacteroides*) that enhance the immune system via Toll-like receptor signaling. LPSs are important because they lead to the maturation of the immune system. However, the prolonged and excessive exposure associated with increased gut permeability can lead to disorders such as insulin resistance, inflammation, and slow growth [27].

Regarding hormonal pathways, such as the growth hormone (GH) insulin-like growth factor-1 (IGF-1) axis, the GM is also involved. Thus, the GM produces linear growth through the action of IGF-1, influencing bone and tissue development through the presence of *Lactobacillus plantarum* species, which restore IGF-1 levels and support normal growth [28,37]. However, the chronic low-grade inflammation produced by factors such as dysbiosis slows the IGF-1 secretion and diverts energy away from anabolic processes. Also, cytokines (e.g., IL-6, TNF-α, and IL-1β) inhibit chondrocyte proliferation and affect bone elongation. In this regard, SCFAs and butyrate attenuate these effects with their anti-inflammatory properties [37].

### 2.2. Gut Microbiota in Neurodevelopmental Disorders and Epilepsy

In recent years, researchers have focused on understanding the influence of the GM in neurodevelopmental disorders through the gut–brain axis. It was observed that an altered GM can result in atypical brain development. Thus, neurodevelopmental psychiatric disorders can be observed from an early age. From the very early stages of fetal development, the initial establishment of the microbiota is synchronized with the development of the nervous system. Also, microbiota maturation corresponds to critical stages of brain development, in which neural connections are highly plastic and potentially susceptible to being affected [20,38]. Any change in this interaction will strongly increase the possibility of neurodevelopmental disorders [39].

**Figure 2:**
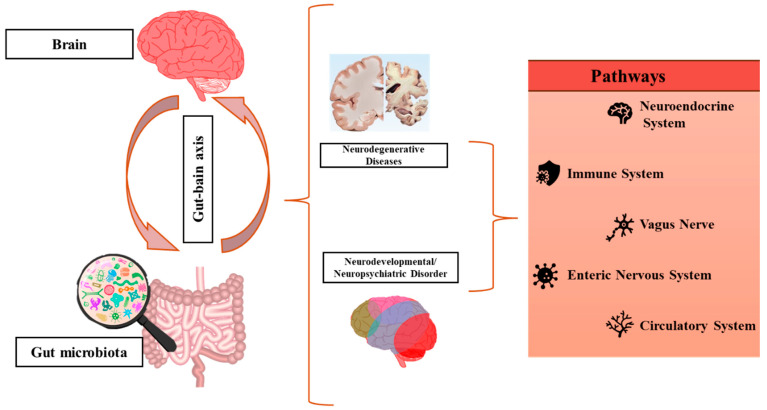
The microbiota–gut–brain axis. Realized based on information from [9,43,44].

The term gut–brain axis (Figure 2) refers to the communication between the central nervous system (CNS) and the gut by the interaction of the immune system, neuronal pathways, and the endocrine/systemic pathway, creating an intricate network [40,41,42]. The enteric nervous system (ENS) in the gut controls and regulates the intestinal metabolism with the help of enteric neurons and neurotransmitters, which transmit information from the gut to the CNS. The vagus nerve represents a communication pathway between the ENS and the CNS, leading to the management of the gut metabolism through the existence of the gut–encephalic axis, through the expression of receptors for gut peptides (e.g., cholecystokinin (CCK), ghrelin, leptin, peptide tyrosine (PYY), glucagon-like peptide-1 (GLP-1), and 5-hydroxytryptamine (5-HT)) [29].

The GM is involved in establishing the blood–brain barrier (BBB), the maturation of microglia, myelination, and neurogenesis. Moreover, the GM is involved in both modulating the production of neurotransmitters, such as noradrenaline, serotonin, dopamine, and histamine, and the production of gamma-aminobutyric acid GABA and their synthesis, which are essential for neural communication [45,46,47]. Additionally, SCFAs are responsible for epigenetic modifications that influence gene expression in the brain [46]. Some factors that influence the CNS plasticity include the production of neuroactive molecules, gene expression, and the modulation of microglial activity [41]. Much of the work in the gut–brain axis has focused on neurodegenerative processes (Alzheimer’s disease, Parkinson’s disease, and Huntington’s disease), which are likely impacted by microbes that are associated with neuroinflammation [47,48].

However, studies are beginning to focus on neurodevelopmental disorders (NDDs). NDDs are defined by various conditions that start to manifest in early childhood and are characterized by the disruption of the natural development of the brain function that influences cognitive, emotional, and motor abilities. These conditions can be associated with complex interactions between genetic and environmental factors that lead to modifications of the brain’s neurodevelopment and neural plasticity. NDDs include Autism Spectrum Disorder, Attention Deficit Hyperactivity Disorder, motor disorders (such as Tourette Syndrome), cerebral palsy, and certain neurogenetic conditions, such as Rett Syndrome, Down Syndrome, Angelman Syndrome, and Turner Syndrome [13,49,50]. Table 1 provides a comparative summary of the main neurodevelopmental disorders that will be discussed in future sections. In this regard, the table compares key aspects of these neurodevelopmental disorders.

#### 2.2.1. Autism Spectrum Disorder

Autism Spectrum Disorder (ASD) represents one of the neurodevelopmental disorders defined by impaired social communication, restricted interests, and repetitive behaviors. Although the exact reasons for the onset of ASD are still unknown, it is thought that a combination of genetic, immunologic, environmental, and GM factors may influence the onset of this condition [66,67,68].

Regarding GM and ASD symptoms, it was observed that they are associated with the gut–brain axis. In this respect, the microbiota influences neurological function by neuroendocrine, neuroimmune, and autonomic nervous pathways [66]. The production of SCFAs, phenol compounds, and free amino acids (FAAs) modulates the function of the brain and influences ASD-related behaviors [66]. However, dysbiosis is common in children with ASD. A study by Chan et al. [69] displayed notable differences in the GM between ASD children and their typically developing (TD) peers. The TD group presented a richer diversity of the GM compared to the ASD group. At the microbial level, the study revealed that certain bacterial classes, like *Deinococci* and *Holophagae*, were significantly lower in the ASD group. Meanwhile, other species, such as *Subdoligranulum* and *Faecalibacterium*, were more prevalent in children with ASD. Furthermore, researchers indicated increased levels of *Proteobacteria*, *Lactobacillus*, *Bacteroides*, *Desulfovibrio*, and *Clostridium*, while beneficial microbes like *Bifidobacterium*, *Blautia*, *Dialister*, *Prevotella*, *Veillonella*, and *Turicibacter* were reduced [13]. Propionic acid (PPA) can be produced by *Clostridia*, *Bacteroidetes*, and *Desulfovibrio* bacteria from the gut, and in high doses, changes in hyperactivity, repetitive behaviors, and dopamine and serotonin neurotransmission were observed [68]. Additionally, in children with ASD, it was observed that an increased permeability of the gut, or “leaky gut”, may increase the risk of the occurrence of bacterial toxins (e.g., lipopolysaccharides (LPSs)), resulting in neuroinflammation and altered brain functions [66]. In this regard, even if propionate has beneficial metabolic roles, its excessive production, together with *Clostridium*-derived endotoxins like p-cresol, may exacerbate ASD symptoms [68].

A study performed by Allan et al. [70] examined the effect of dietary changes in children with ASD. The focus was on how a modified ketogenic diet produces changes in the microbiota, inflammatory markers, and microRNAs associated with brain function. It was observed that the most significant finding was the increase in the diversity of the children’s gut microbiomes, with notable changes across various levels (e.g., a rise in *Lactobacillale* and a decrease in Bacteroides and *Ruminococcus*), including the family and species. Also, the diet increased the expression of butyrate kinase, a SCFA involved in maintaining gut health that reduces neuroinflammation, which is often a concern for individuals with ASD. The study also highlighted the considerable reduction in pro-inflammatory cytokines (e.g., IL-1β and IL-12p70). On the other hand, levels of the brain-derived neurotrophic factor (BDNF), which is essential for brain development and function, dropped noticeably, suggesting a complex interaction between diet and neuroinflammation.

At this moment, researchers are focusing on probiotics (beneficial bacteria) as an alternative treatment to alleviate GI symptoms and possibly improve anxiety and behavioral symptoms in children with ASD. The administration of probiotics and prebiotics can help restore the GM balance and may improve ASD symptoms. A study performed by Wang et al. [71] investigated the effects of a probiotic and fructo-oligosaccharide (FOS) intervention on ASD symptoms. The research team examined the changes in the GM, SCFAs, and neurotransmitter levels. In the discovery phase of the study, findings suggested that children with ASD had reduced beneficial bacteria (e.g., *Bifidobacterium longum* and *Bifidobacteriales*), while harmful bacteria such as *Clostridium* and *Ruminococcus*, associated with inflammation and gut permeability issues, had increased levels. Additionally, it was observed that children with ASD had lower levels of microbial richness and diversity compared to TD children. SCFA levels are observed to be at lower levels in children with children, which suggests an impaired gut microbial metabolism. Regarding neurotransmitter imbalances, Wang et al. [71], noticed increased serotonin (5-HT) and its metabolite 5-hydroxyindoleacetic acid (5-HIAA) in plasma, while homovanillic acid (HVA), a dopamine metabolite, is found in lower concentrations. Moreover, lower kynurenine levels indicate an imbalance in the serotonin/kynurenine pathway. These imbalances might be associated with ASD behavioral symptoms. In the interventional phase, Wang et al. [71] administrated children with ASD with a probiotic mixture (*Bifidobacterium infantis*, *Lactobacillus rhamnosus*, *Bifidobacterium lactis*, and *Lactobacillus paracasei*) together with fructo-oligosaccharide (FOS), while the other ASD group received a maltodextrin placebo. Researchers observed an improvement in the GM in children with ASD from the probiotics + FOS group (increased beneficial bacteria, reduced harmful bacteria, and restored microbial diversity). In contrast, in the placebo group, there were no such changes. The administration of the probiotics + FOS treatment showed a significant increase in acetic acid, propionic acid, and butyric acid, suggesting a restored gut microbial function and an enhanced SCFA production. Additionally, the probiotics + FOS intervention reduced hyperserotonemia and improved the dopamine metabolism. The behavioral symptoms of autism were evaluated. The study provides evidence that the ASD severity significantly decreased. Speech, communication, sociability, sensory, and cognitive awareness improvements were observed. No improvements were seen in the placebo group, confirming the effectiveness of probiotics + FOS.

Kong et al. [72] performed a randomized, double-blind, and placebo-controlled study that combined therapy with probiotics (*Lactobacillus plantarum* PS128) and oxytocin (OXT), which exerts beneficial effects on ASD symptoms. Subjects were randomized into two groups: Phase 1—oral placebo and oral probiotics and Phase 2—intranasal (OXT) + oral placebo and intranasal OXT + oral probiotics. The probiotics–OXT therapy showed promising effects in improving social behaviors, cognition, and gut health in individuals with ASD. Also, the combination therapy increased the microbiome diversity and enhanced favorable bacterial interactions. These results suggest that GM modulation might be a mechanism behind the behavioral improvements.

Sanctuary et al. [73] wanted to evaluate the safety, tolerability, and potential effects of a probiotic (*Bifidobacterium infantis*) combined with a bovine colostrum product (BCP) (prebiotic) in children with ASD and GI symptoms. The participants were divided into two groups: BCP + probiotics and BPC alone (prebiotic). GI symptoms improved in both therapies, with significant improvements in pain with bowel movements, diarrhea frequency, and stool consistency. However, after stopping the supplement, seven out of eight children experienced a return of their GI symptoms. Behavioral improvements (irritability, hyperactivity, and repetitive behaviors) were observed, particularly in the BCP group. A reduction in pro-inflammatory markers (IL-13 and TNF-α) was observed, suggesting a potential immune-modulating effect of the treatment. GM changes were minimal, but certain bacterial shifts correlated with symptom improvements.

Hrnciarova et al. [74] investigated whether a Juvenil supplementation can modify the GM in children with ASD and if it can improve their symptoms. Juvenil represents a nontoxic alcohol–ether extract of a bovine tissue supplement with a modulatory activity on immunity, dominantly on cells of the innate immune system and the organism’s regeneration. At the beginning of the study, it was observed that there were differences between the ASD group and the TD group’s microbiota. After three months of the Juvenil supplementation, the microbiota of the children with ASD in the Juvenil group was no longer significantly different from that of the TD children. However, the children with ASD’s microbiota shifted to a more TD-like profile but did not fully restore to a complete TD state. Regarding the symptoms of the children with ASD, the Juvenil administration enhanced symptoms such as their motor function, visual reactions, fear and nervousness, nonverbal communication, and activity level.

#### 2.2.2. Attention Deficit Hyperactivity Disorder

Attention Deficit Hyperactivity Disorder (ADHD) represents a prevalent neurodevelopmental disorder, characterized by inattention, hyperactivity, and impulsivity. This condition persists in adolescence and adulthood, representing a significant global burden [75,76]. Currently, researchers have started to evaluate the potential link between the GM composition and ADHD symptoms. The findings suggested that microbial imbalances can be correlated with hyperactivity and attention deficits [13]. Some findings indicate that individuals diagnosed with ADHD often experience GI dysfunction, including digestive issues, low-grade inflammation, and constipation, highlighting the fact that the GM might be involved in the ADHD pathology [77]. Thus, it was observed that children with ADHD have deficiencies in SCFAs [77]. It is well known that connections between neuroactive metabolites produced by the GM neurotransmitter production and neuropsychiatric disorders are linked [77,78,79]. Cickovski et al. [77] performed a study on rodents, exploring their anxiety and social behavior, which are linked to the alteration of the hippocampal and amygdala neurotransmission driven by the gut microbiome composition. In this regard, some clinical trials have started to examine the potential effect of modulating the GM on ADHD symptoms.

Ast et al. [80] carried out an 8-week randomized controlled trial (RCT) that evaluated the effect of the micronutrient intake vs. placebos in children with ADHD. The study was focused on finding whether changes in the GM were associated with behavioral improvements and whether specific microbial changes could be linked to an ADHD symptom response. After the micronutrient intake, significant microbial composition changes were observed, such as in the bacterial richness and evenness, compared to the placebo group. A promising sign of improving the gut microbiome in children with ADHD is the significant decrease in *Actinobacterium*, which is generally abundant in ADHD patients. Also, bacteria responsible for butyrate production, such as *Oscillospiraceae* and *Rikenellaceae*, increased significantly, which has been related to enhanced neurotransmitter activity and decreased inflammation, both of which are important in ADHD. *Bacteroidota* increased, while *Firmicutes* decreased, showing a shift in the overall microbiome balance. These findings suggest that the microbiome composition may influence ADHD symptoms and that a micronutrient supplementation could modulate the GM. Another similar study was performed by Stevens et al. [81], which aimed to examine the impact of a broad-spectrum micronutrient supplementation on the gut microbiome, if micronutrient treatments can produce changes in the microbiota composition, and whether these changes are associated with visible improvements in children with ADHD. Thus, small but specific changes in the microbiota composition were observed (decrease in Bifidobacterium levels and increased levels of Collinsella species), and this can suggest that micronutrients may enhance microbiota diversity. Moreover, ADHD symptom scores improved in some children receiving the treatment with micronutrients. Decreased levels of Actinobacteria species (e.g., *Bifidobacterium*) might explain the symptom improvement. So, a micronutrient supplementation could be a treatment strategy that, even if it offers small changes in the gut microbiome, is important to improve ADHD symptoms in children.

Wang et al. [82] undertook a 12-week randomized, double-blind, placebo-controlled trial which evaluated how *Bifidobacterium bifidum* (Bf-688) affects the ADHD symptoms, neuropsychological performance, body weight, and gut microbiome composition. Results showed that the administration of Bf-688 improved visual and auditory attention, reduced omission errors, and resulted in faster reaction times. Moreover, Bf-688 contributed to an increase in the *Firmicutes*/*Bacteroidetes* ratio, which enhanced the gut barrier function and neurotransmitter regulation, improving dopamine precursor synthesis and modulating dopamine-related cognitive functions. Bf-688 reduced GI symptoms, such as stomach aches and the loss of appetite.

#### 2.2.3. Tic Disorders and Tourette Syndrome

Tourette Syndrome (TS) represents a common neurodevelopmental disorder outlined by multiple motor and at least one vocal/phonic tic. These symptoms generally persist for at least one year and start to appear in childhood when this condition is diagnosed [13,83]. In general, this syndrome is associated with other neurodevelopmental conditions, such as ADHD and Obsessive–Compulsive Disorder (OCD), affecting approximately 90% of patients [83,84]. TS is managed using pharmacotherapy, which includes dopamine-modulating agents, alpha-2-adrenergic agonists, and atypical neuroleptics, such as haloperidol, aripiprazole, risperidone, and tiapride, and other treatment strategies such as psychotherapy and neurosurgical interventions, particularly deep brain stimulation (DBS), which may be considered for severe cases [83,85]. Recent studies indicate the link between GM imbalances and the severity of TS symptoms. In patients diagnosed with TS, levels of *Ruminococcaceae* and *Bacteroides* are increased, being responsible for immune activation and neuroinflammation. Additionally, decreased levels of *Firmicutes* and increased levels of Proteobacteria present a shift in the microbial balance that may influence tic severity [85].

Wu et al. [86] performed a randomized controlled trial in which probiotic Lactobacillus plantarum (PS128) was administered to children with TS. The study aimed to evaluate the effects of the probiotic strain *Lactobacillus plantarum* PS128 on the tic severity and common comorbidities of TS in children. Researchers observed improvements in tic severity over two months, significant changes in attention deficits, and better attention and impulse control.

Liang et al. [87] explored the effectiveness of probiotics (*Limosilactobacillus reuteri* CGMCC No. 25664), hypothesizing that their clinical efficacy is comparable to that of clonidine in treating chronic Tic Disorders. After eight weeks of administration, the severity of the tic decreased significantly, with an improvement in attention-related issues and a greater reduction in hyperactivity symptoms.

#### 2.2.4. Down Syndrome

Down Syndrome (DS) represents one of the most well-known chromosomal disorders caused by the trisomy of chromosome 21. This condition is associated with a high range of clinical manifestations, including intellectual disability and musculoskeletal, neurological, and cardiovascular conditions. Individuals diagnosed with DS present common physical and developmental features, including a short stature, muscle hypotonia, intellectual disability (ranging from mild to severe), cerebellar hypoplasia, and congenital heart defects, especially atrioventricular septal defects [64,88]. Moreover, individuals with DS have a high prevalence of medical comorbidities, such as recurrent infections, hypothyroidism, autoimmune diseases, epilepsy, vision and hearing impairments, hematological disorders (including leukemia), early-onset Alzheimer’s disease, and various psychiatric or behavioral conditions like anxiety disorders [64]. Recent studies focus on the gut–brain axis in DS because significant alterations are observed in the GM. Children with reduced microbiota and a decrease in the family of *Acidaminococcaceae* can be associated with a low concentration of fecal propionate [89,90]. Additionally, studies showed that certain bacterial strains in the gut correlate with cognitive function scores, which indicates a link between the microbiome composition and neurological outcomes in children with DS [13].

#### 2.2.5. Epilepsy

Epilepsy, affecting over 65 million people worldwide, is a group of heterogeneous neurological conditions defined by an enduring predisposition to generate seizures [91,92]. Despite advances in treatments, around 30% of patients remain resistant to conventional antiseizure medication, underscoring the urgent need for new therapeutic targets, biomarkers, and a deeper understanding of epileptogenesis [93,94].

One such promising area of research is the gut microbiota—this complex ecosystem has been increasingly recognized as a critical modulator of the brain development and function via the microbiota–gut–brain axis, which involves intricate neural, immune, metabolic, and endocrine pathways. Recent preclinical and clinical studies suggest that alterations in the gut microbiota composition may influence key mechanisms of epileptogenesis, including neuroinflammation, neurotransmitter release, neuronal hyperexcitability, and neural network remodeling. Indeed, epilepsy-associated changes in the microbiota composition have been reported both in patients and in animal models, suggesting that these microbial shifts are not merely coincidental but potentially contributory [95].

Furthermore, some research indicates that gut microbiota profiles may help distinguish drug-responsive from drug-resistant epilepsy cases or even predict the susceptibility to epilepsy following neurological insults. However, the exploration of the microbiota as a biomarker remains in its early stages, limited by small sample sizes, heterogeneous populations, and a lack of methodological standardization [95,96].

Perhaps most compelling is the emerging evidence supporting the therapeutic potential of manipulating the microbiota, including through cannabidiol (CBD). As a compound with known anti-inflammatory and neuromodulatory effects, CBD may act, in part, via the gut microbiota, though its mechanisms are still being unraveled. Some studies have begun to explore how CBD alters the gut ecosystem, possibly contributing to its antiseizure properties [97,98].

Although the concept of a link between the gut and seizures dates back to early 20th-century hypotheses, such as the idea of a “*Bacillus epilepticus*”, only in the last two decades has progress in genomic and sequencing technology enabled the investigation of these connections rigorously. Today, the bidirectional communication between gut microbes and the brain is increasingly viewed as a dynamic system that can influence not only epilepsy risk but also treatment outcomes [95,99].

#### 2.2.6. Clinical Studies

**Figure 3:**
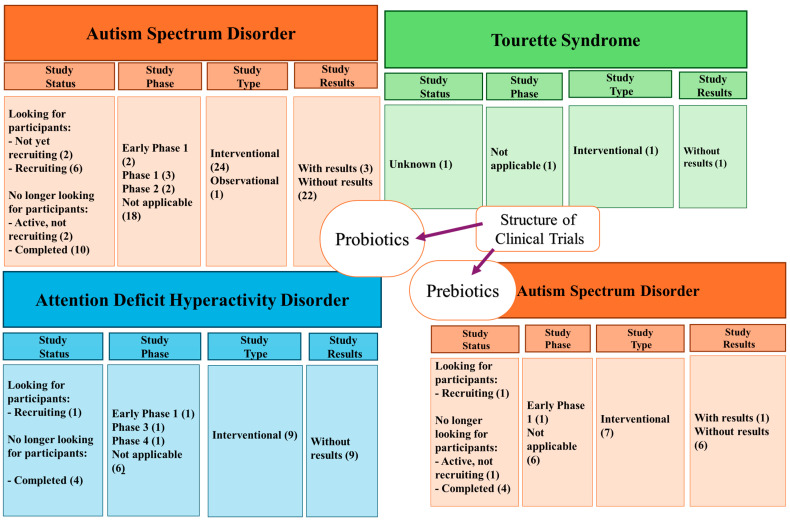
The structure of clinical studies available on ClinicalTrials.gov as of May 2025.

Although we can see an improvement in the symptoms of neurodevelopmental diseases in the studies presented above, genetic diseases with poor outcomes, such as Down Syndrome and others, cannot be fundamentally solved by manipulating the microbiota. Regarding the clinical trials (Figure 3) realized with the administration of probiotics present on ClinicalTrials.gov, the following can be observed: there are 25 studies realized for ASD, 1 study for TS, for ADHD, there are 9 studies, and for DS, no studies are reported. On the other hand, there are seven clinical trials on administering prebiotics in patients with ASD. The analysis of the existing clinical trials shows an increased interest in probiotics and prebiotics in autism (ASD), while other conditions, such as ADHD, Tourette’s, and SD, are much less investigated.

### 2.3. Treatment Strategies for Gut-Related Disorders

In terms of treatment strategies for intestinal microbiota-related diseases, prebiotics, probiotics, and fibers are presented as an alternative to improve symptoms. Thus, with this strategy, improvements in the production of SCFAs and a decrease in inflammation have been observed. Probiotics containing *Lactobacillus* and *Bifidobacterium* strains led to improved intestinal permeability and reduced inflammation, and, in the case of obesity, it led to decreased fat and improved patients’ lipid profiles. The same effects were observed with prebiotics [31,100].

Additionally, the treatment strategy for neurodevelopmental disorders is challenging. For example, individuals diagnosed with ASD are more vulnerable to the side effects of psychopharmacological agents [101,102]. Children with ASD are reported to receive psychopharmacological interventions, such as stimulants, antidepressants, alpha-2 agonists, antipsychotics, and anticonvulsants [102,103]. Regarding children with ADHD, the most widely used medications are methylphenidate (MPH), amphetamines (AMPs), atomoxetine (ATX), guanfacine (GFC), clonidine (CLO), bupropion, modafinil, and tricyclic antidepressants (TCAs). These therapies are usually prescribed but have considerable side effects, such as a lack of response or intolerance [104]. At the same time, previous sections have highlighted the impact of using prebiotics, probiotics, and fibers in improving the symptoms of neurodevelopmental disorders in children. However, probiotics and prebiotics have some disadvantages. Prebiotic (e.g., oligosaccharides and polysaccharides) side effects are associated with diarrhea, bloating, cramps, and flatulence, while concerns about probiotics are mainly about their safety. Probiotics can interact with commensal bacteria. They can also directly impact the host, generating and releasing various toxic metabolites, causing metabolic disturbances and food poisoning [82,105].

## 3. Liposomal and Lipid-Based Drug Delivery Systems in Pediatric Disorders

### 3.1. Mechanisms of Action

#### 3.1.1. Liposome-Based Drug Delivery Systems

**Figure 4:**
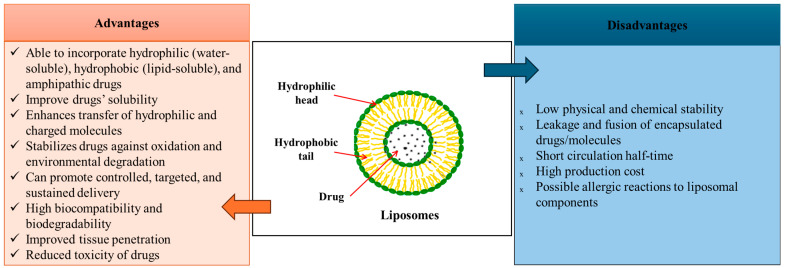
Liposomes: structure, advantages, and disadvantages. Based on information from [118,119].

Liposomes (Figure 4) are spherical vesicles synthesized by hydrating dry phospholipids. They form an aqueous core surrounded by lipid bilayers, with a unique structure that allows hydrophilic compounds to be encapsulated in the core, while the hydrophobic compounds are within the lipidic layer, making them an excellent alternative for highly effective controlled drug delivery systems [106,107,108,109,110,111]. They are relatively easy to produce in large quantities, and their physicochemical properties, such as their surface area, size, and lipid composition, can be manipulated to optimize the drug delivery system [112]. Thus, lipid formulations generally provide increased drug solubilization for water-insoluble drugs. In this sense, the drug can be dissolved in the lipid matrix, and its absorption is better than conventional solid dosage forms [113,114]. Liposomes can be classified based on their composition, structure, and size [106,107,108,109,110]. Liposomes’ sizes can vary from 0.025 to 2.5 µm [109]. Their simplest structure consists of a single lipid bilayer enclosing an aqueous compartment that allows drug loading, while complex structures are represented by multilamellar liposomes. Multilamellar liposomes contain multiple lipid layers that are separated by aqueous spaces. Their flexible structure promotes the simultaneous encapsulation of multiple drugs, enhancing biocompatibility, bioavailability, and therapeutic efficacy while minimizing toxicity. Thus, their composition gives them advantages such as the ability to fuse with cell membranes, enabling the direct release of therapeutic agents into the cytoplasm. Also, liposomes can be used to create targeted delivery systems that transport antioxidants, mitochondrial DNA (mtDNA), and other molecules directed toward the mitochondria [106,107,115,116]. In this respect, the number of lipid layers, composition, and overall size are critical factors influencing liposomes’ circulation, half-life, and drug encapsulation capacity [108,109,117].

However, even if liposomes have great advantages, some challenges regarding their stability and storage need to be overcome. In an aqueous solution, they are predisposed to drug leakage and aggregation over time, which can reduce their effectiveness. Also, their composition, chain length, and cholesterol content represent a key factor in their stability due to the fragile bilayer phospholipid membranes and oxidation or hydrolysis of the fatty acids [109,110]. The cholesterol ratio is important for liposome integrity, reinforcing the bilayer, increasing membrane rigidity, and decreasing their permeability. Without cholesterol, liposomes tend to be more fluid and unstable and susceptible to premature drug release and structural breakdown. To combat some of these disadvantages, techniques such as lyophilization, spray drying, and supercritical fluid processing are used to store liposomes in a solid form to extend their shelf life. Also, by modifying the cholesterol ratio, liposomes can be obtained with an improved performance for different therapeutic applications [109,120]. Physiochemical stability can be improved by adding polysaccharides during the liposome preparation, which can minimize the oxidative degradation of liposomes, including high-quality lecithin with low levels of hydroperoxides [110].

Liposomes can be administered through various routes, including oral, topical, nasal, transdermal, and direct brain delivery. However, a limitation of liposome administrations is represented by their quick metabolization by the reticuloendothelial system (RES), especially in the spleen and liver, and the action of plasma proteins that recognize and mark liposomes for elimination, resulting in a short time in the circulation. In this regard, the PEGylation of liposomes can enhance their detectability by the immune system and prolong their half-life in the bloodstream [121]. Orally administered liposomes must get past the gastrointestinal barrier, including high pH levels, enzyme breakdown, and the mucus layer, which might slow the absorption rate. Recent improvements in lipid-based formulations have focused on the intestinal lymphatic system to improve the effectiveness of drug absorption and distribution [122,123].

Liposomal-based drug delivery systems involve several steps, leading to more efficient drug delivery. Drug encapsulation is an important mechanism that involves the drug’s enclosure in the core of the lipid bilayer, which is intended to protect it from breaking down in the body’s fluids. In time, the liposomes’ bilayer is degraded, promoting a controlled release of the drug content [124]. The main liposomal drug action is represented by adsorption, which facilitates the drug transfer into the cell [112]. In this respect, there is a specific interaction with cell surface components in which liposomal-based drug delivery systems’ lipidic bilayer fuses with cellular membranes, and the drug release occurs into the cell or nearby. Also, lipids can be exchanged with cell membranes without liposomes’ complete internalization. Additionally, these carriers can bind to specific receptors on the cell surface, promoting the liposomes’ internalization into them [112,125]. Other mechanisms through which liposomes penetrate inside cells are represented by endocytosis and pinocytosis. In endocytosis, the cells surround the drug carrier, allowing the drug to be transported inside the cell, while through pinocytosis, in the extracellular fluid and together with liposomes, they form small vesicles that are absorbed into cells [112,125,126].

The specific targeting is a primordial functional property of liposomes as drug delivery systems. Active targeting represents a surface modification of liposomes to ensure that they bind to target cells, tissues, or organs (e.g., GM). This strategy makes them more efficient in drug administration by reducing side effects and promoting improved therapeutic outcomes. Active targeting is achieved by modifying the surface of liposomes with specific molecules, such as antibodies, peptides, folate, and aptamers, to optimize targeting strategies and overcome regulatory barriers [127,128].

#### 3.1.2. Solid Lipid-Based Drug Delivery Systems

**Figure 5:**
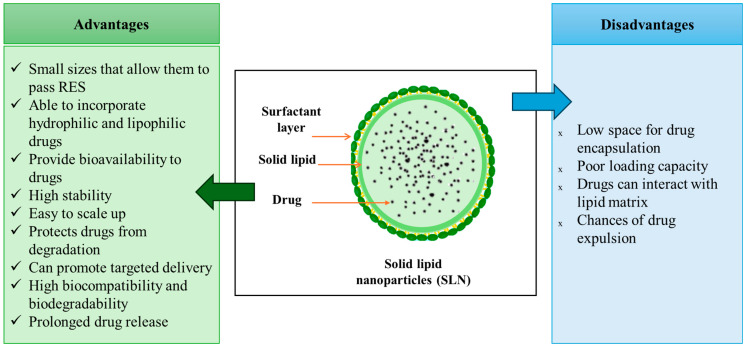
SLN: structure, advantages, and disadvantages. Based on information from [134,135].

Solid lipid nanoparticles (SLNs) (Figure 5) represent spherical, biocompatible nanocarriers between 50 and 1000 nm in size. SLN formulations comprise solid-state lipids, emulsifiers, and sometimes a mixture of active pharmaceutical ingredients (APIs) and an adequate solvent system [129,130,131,132]. The types of solid lipids used in SLNs’ manufacturing are triglycerides, partial glycerides, free fatty acids, steroids, and waxes [133]. These systems have excellent advantages (Figure 5) and, in this regard, are developed to overcome other drug delivery systems’ limitations, such as polymer degradation, drug leakage, and cytotoxicity [129,130,131].

Thus, their biocompatibility and physicochemical diversity make them an alternative drug delivery system that enhances drug bioavailability [136]. SLNs comprise a hydrophobic lipid core in which hydrophilic and lipophilic drugs can be dispersed [131]. Their solid matrix enhances the control of the drug release by stabilizing their chemical biodegradation, protecting the drug [131,137]. SLNs are preferable in their use as controlled-release delivery systems in the gastrointestinal tract, as they enhance the controlled release of lipid-enclosed active substances [133]. Thus, SLNs can improve drug absorption by different pathways, such as by increasing the membrane permeability and intestinal drug dilution, inhibiting the P-glycoprotein (P-gp) efflux transporters, reducing cytochrome P450s (CYPs)’s enzymatic activity, and increasing chylomicron biosynthesis and the lymphatic transport rate [136]. Moreover, because of the dense matrix, the digestion of lipids is slowed down, allowing drug release in a more long-lasting manner [133]. Their surface can be easily modified, which makes their time in the systemic circulation much longer, and thus, their pharmacokinetic profile is improved [138]. In the same manner as liposomes, SLNs interact with proteins at the plastid level after their absorption, forming a protein corona (PC), which alters their biological identity, thus changing their distribution, clearance, and immune recognition. In addition, surface charge increases and the lack of surface modifications (e.g., PEGylation) accelerates the mononuclear phagocyte system (MPS) uptake, limiting the circulation time [139].

### 3.2. Potential Use of Liposomes and Lipid-Based Drug Delivery Systems in Pediatric Gut-Related Disorders

The administration of medicines for children remains challenging, as most medicines are only present in solid dosage forms (e.g., tablets and capsules), making pediatric administration difficult. Some drugs are usually administered by dividing adult pharmaceutical formulations into smaller doses, which can lead to inaccurate dosing and poor medication adherence, implying undesirable adverse effects or treatment inefficacy [140]. Furthermore, some medicines’ taste is unpleasant, and children tend to spit them out. So, in this regard, a sweetened formulation with easier weight-based dosing will be better tolerated by them [140,141,142]. Besides other advantages already discussed, lipid-based drug delivery systems seem to be an alternative for developing a rapidly dissolvable and flexible solid pediatric formulation that presents the possibility of a drug encapsulation that masks the drug’s taste [141]. Research has focused on obtaining efficient drug delivery systems for pediatric use. Thus, it has been demonstrated through clinical trials registered on ClinicalTrials.gov that the use of lipid-based drug delivery systems is safe for the administration of active substances, such as mitoxantrone, irinotecan, iron, daunorubicin, and cytarabine, in children [143,144,145,146,147].

In terms of treating conditions related to the intestinal microbiota, it has been observed that using strategies to modulate it yields results that improve symptoms in children. In this regard, liposomes could deliver probiotics and prebiotics, improving symptoms associated with the GM and neurodevelopmental diseases. In a study by Cao et al. [148], researchers created a biocompatible lipid membrane, thus forming a lipid-coated bacterium (LCB), which showed a three times higher survival rate in the stomach and four times higher bioavailability in the gut than uncoated *Escherichia coli* (*E. coli*). It was observed that lipid membranes dissolve gradually in the gut environment, allowing the bacteria to adhere and colonize the gut mucosa. In the same manner, Chowdhuri et al. [149] focused on the encapsulation of living cells (*E. coli*) inside liposomes (giant unilamellar vesicles (GUVs)) using the inverse-emulsion technique.

Azeem et al. [150] developed solid lipid microparticles (SLMPs) using two wall materials—whey protein isolate (SLMW) and gum Arabic (SLMG)—to encapsulate probiotics (*Lactobacillus rhamnosus GG*). The study aimed to evaluate the viability; encapsulation efficiency; morphological and molecular structure; and survival under stressed conditions, like gastric/intestinal fluid, heat, and storage. The final product of this study was probiotic chocolate. It was observed that the SLMP maintained its viability under gastric acid, intestinal enzymes, heat, and storage stress. Also, Kumar et al. [151] obtained chitosan-solid lipid nanoparticles (SLNs) for a probiotic Lactobacillus plantarum encapsulation to improve its stability in harsh GI conditions. It was observed that the encapsulation method protected probiotic cells from GI factors, such as acidic gastric fluids, bile salts, and enzymatic degradation, with a viability that remained consistent over time, especially under GI conditions. The same results were observed in Han et al. [152], who confirm that single-cell encapsulation using a layer-by-layer (LbL) technique is a highly promising mechanism for protecting probiotics such as *E. coli* Nissle.

A clinical trial performed by Apte et al. [63] evaluated if a daily infant body massage with liposomal micronutrient-fortified (LMF) oil containing vitamin D, Iron, folate, and vitamin B12 can improve neurodevelopment and prevent vitamin D deficiency and anemia. It was observed that infants tolerated the administration of the LMF oil well and it was safe to use, improving the vitamin D status, while no significant changes were observed against anemia overall. Some benefits were observed in moderately anemic infants. Regarding the neurodevelopmental changes, slight motor and social benefits appeared in specific subgroups.

## 4. Conclusions

Considering that GM is involved in a multitude of physiological processes, dysbiosis may be a key factor in worsening symptoms of neurodevelopmental disorders. Based on the studies presented in this paper, the research supports the hypothesis of a link between the GM and symptoms of neurodevelopmental disorders in children. Thus, the administration of drugs that stimulate and restore the gut microbiota (e.g., probiotics and prebiotics) may have the potential to ameliorate some of their symptoms. On the other hand, liposomes and solid lipid nanoparticles are versatile drug delivery systems with remarkable drug delivery properties due to their unique structures that allow the incorporation of both hydrophilic and hydrophobic substances. They are preferred in drug delivery due to their improved drug solubility, increased bioavailability, and reduced adverse effects of active substances.

However, drug delivery to children remains a challenge, and lipid-based systems can provide solutions to mask unpleasant tastes and formulations to allow proper dosing and improve the adherence to treatments. Liposomes and solid lipid nanoparticles have been safely used in children’s treatments and in the administration of probiotics, prebiotics, and immunomodulators for the GM, with promising results in improving children’s neurodevelopment and quality of life.

## Figures and Tables

**Table 1:** Overview of neurodevelopmental disorders.

Disease	Estimated Prevalence	Comorbidities	Refs.
Autism Spectrum Disorder (ASD)	About 3.2% of children aged 8 years have been identified with ASD ASD is over 3 times more common among boys than among girls.	Intellectual Disability, Anxiety Depression Mood Disorders, Sleep Disorders Epilepsy Metabolic Disorders Immune Dysfunction GI Disorders	[51,52,53,54]
Attention Deficit Hyperactivity Disorder (ADHD)	5% of children and adolescents are affected by ADHD Boys are more likely to be diagnosed with ADHD than girls About 6 in 10 children can present moderate or severe ADHD	ASD Tic Disorders Learning Disorders Depression Bipolar Disorder Anxiety Conduct disorder	[55,56,57,58]
Tic Disorders and Tourette Syndrome	1% of the population	Attention Deficit Hyperactivity disorder (ADHD), Obsessive–compulsive disorder (OCD) Obsessive–compulsive behavior (OCB) Depression Anxiety Rage attacks Self-injurious behavior (SIB)	[59,60,61,62]
Down Syndrome	Each day, 3000 to 5000 children are born with DS	Cerebellar Hypoplasia Anxiety Depression Epilepsy Congenital Heart Defects Immune Dysfunction Obesity Bowel Dysfunction Autoimmune Diseases	[63,64,65]

## References

[1] Rinninella E., Raoul P., Cintoni M., Franceschi F., Miggiano G.A., Gasbarrini A., Mele M.C. (2019). What Is the Healthy Gut Microbiota Composition? A Changing Ecosystem across Age, Environment, Diet, and Diseases. Microorganisms.

[2] Thursby E., Juge N. (2017). Introduction to the human gut microbiota. Biochem. J..

[3] Binda C., Lopetuso L.R., Rizzatti G., Gibiino G., Cennamo V., Gasbarrini A. (2018). Actinobacteria: A relevant minority for the maintenance of gut homeostasis. Dig. Liver Dis..

[4] Sánchez B., Ruiz L., Gueimonde M., Ruas-Madiedo P., Margolles A. (2013). Adaptation of bifidobacteria to the gastrointestinal tract and functional consequences. Pharmacol. Res..

[5] Patangia D.V., Anthony Ryan C., Dempsey E., Paul Ross R., Stanton C. (2022). Impact of antibiotics on the human microbiome and consequences for host health. MicrobiologyOpen.

[6] Lathakumari R.H., Vajravelu L.K., Satheesan A., Ravi S., Thulukanam J. (2024). Antibiotics and the gut microbiome: Understanding the impact on human health. Med. Microecol..

[7] Li X., Watanabe K., Kimura I. (2017). Gut microbiota dysbiosis drives and implies novel therapeutic strategies for diabetes mellitus and related metabolic diseases. Front. Immunol..

[8] Abdukhakimova D., Dossybayeva K., Poddighe D. (2021). Fecal and Duodenal Microbiota in Pediatric Celiac Disease. Front. Pediatr..

[9] Saeed N.K., Al-Beltagi M., Bediwy A.S., El-Sawaf Y., Toema O. (2022). Gut microbiota in various childhood disorders: Implication and indications. World J. Gastroenterol..

[10] Gacesa R., Kurilshikov A., Vich Vila A., Sinha T., Klaassen M.A.Y., Bolte L.A., Andreu-Sánchez S., Chen L., Collij V., Hu S. (2022). Environmental factors shaping the gut microbiome in a Dutch population. Nature.

[11] Bucurica S., Lupanciuc M., Ionita-Radu F., Stefan I., Munteanu A.E., Anghel D., Jinga M., Gaman E.L. (2023). Estrobolome and Hepatocellular Adenomas—Connecting the Dots of the Gut Microbial β-Glucuronidase Pathway as a Metabolic Link. Int. J. Mol. Sci..

[12] Mani S., Boelsterli U.A., Redinbo M.R. (2014). Understanding and modulating mammalian-microbial communication for improved human health. Annu. Rev. Pharmacol. Toxicol..

[13] Borrego-Ruiz A., Borrego J.J. (2024). Neurodevelopmental Disorders Associated with Gut Microbiome Dysbiosis in Children. Children.

[14] Akagawa S., Akagawa Y., Yamanouchi S., Kimata T., Tsuji S., Kaneko K. (2021). Development of the gut microbiota and dysbiosis in children. Biosci. Microbiota Food Health.

[15] Iddrisu I., Monteagudo-Mera A., Poveda C., Pyle S., Shahzad M., Andrews S., Walton G.E. (2021). Malnutrition and Gut Microbiota in Children. Nutrients.

[16] Derrien M., Alvarez A.-S., de Vos W.M. (2019). The Gut Microbiota in the First Decade of Life. Trends Microbiol..

[17] Schwartz D.J., Langdon A.E., Dantas G. (2020). Understanding the impact of antibiotic perturbation on the human microbiome. Genome Med..

[18] Su Q., Liu Q. (2021). Factors affecting gut microbiome in daily diet. Front. Nutr..

[19] Huang H., Jiang J., Wang X., Jiang K., Cao H. (2024). Exposure to prescribed medication in early life and impacts on gut microbiota and disease development. eClinicalMedicine.

[20] Ronan V., Yeasin R., Claud E.C. (2021). Childhood Development and the Microbiome—The Intestinal Microbiota in Maintenance of Health and Development of Disease During Childhood Development. Gastroenterology.

[21] Ihekweazu F.D., Versalovic J. (2018). Development of the Pediatric Gut Microbiome: Impact on Health and Disease. Am. J. Med. Sci..

[22] Meng X., Zhang G., Cao H., Yu D., Fang X., de Vos W.M., Wu H. (2020). Gut dysbacteriosis and intestinal disease: Mechanism and treatment. J. Appl. Microbiol..

[23] Malkawi W.A., AlRafayah E., AlHazabreh M., AbuLaila S., Al-Ghananeem A.M. (2022). Formulation Challenges and Strategies to Develop Pediatric Dosage Forms. Children.

[24] Nieto González N., Obinu A., Rassu G., Giunchedi P., Gavini E. (2021). Polymeric and Lipid Nanoparticles: Which Applications in Pediatrics?. Pharmaceutics.

[25] Marques M.S., Lima L.A., Poletto F., Contri R.V., Kulkamp Guerreiro I.C. (2022). Nanotechnology for the treatment of paediatric diseases: A review. J. Drug Deliv. Sci. Technol..

[26] Ivanovska V., Rademaker C.M.A., van Dijk L., Mantel-Teeuwisse A.K. (2014). Pediatric Drug Formulations: A Review of Challenges and Progress. Pediatrics.

[27] Durda-Masny M., Ciomborowska-Basheer J., Makałowska I., Szwed A. (2022). The Mediating Role of the Gut Microbiota in the Physical Growth of Children. Life.

[28] He P., Shen X., Guo S. (2023). Intestinal flora and linear growth in children. Front. Pediatr..

[29] Wang S.-Z., Yu Y.-J., Adeli K. (2020). Role of Gut Microbiota in Neuroendocrine Regulation of Carbohydrate and Lipid Metabolism via the Microbiota-Gut-Brain-Liver Axis. Microorganisms.

[30] Rowland I., Gibson G., Heinken A., Scott K., Swann J., Thiele I., Tuohy K. (2018). Gut microbiota functions: Metabolism of nutrients and other food components. Eur. J. Nutr..

[31] John G.K., Mullin G.E. (2016). The Gut Microbiome and Obesity. Curr. Oncol. Rep..

[32] Luo Y., Li M., Luo D., Tang B. (2025). Gut Microbiota: An Important Participant in Childhood Obesity. Adv. Nutr..

[33] Alcazar M., Escribano J., Ferré N., Closa-Monasterolo R., Selma-Royo M., Feliu A., Castillejo G., Luque V., Closa-Monasterolo R., Escribano J. (2022). Gut microbiota is associated with metabolic health in children with obesity. Clin. Nutr..

[34] Bartlett A., Kleiner M. (2022). Dietary protein and the intestinal microbiota: An understudied relationship. iScience.

[35] Richard D.M., Dawes M.A., Mathias C.W., Acheson A., Hill-Kapturczak N., Dougherty D.M. (2009). L-Tryptophan: Basic Metabolic Functions, Behavioral Research and Therapeutic Indications. Int. J. Tryptophan Res. IJTR.

[36] Jian C., Carpén N., Helve O., de Vos W.M., Korpela K., Salonen A. (2021). Early-life gut microbiota and its connection to metabolic health in children: Perspective on ecological drivers and need for quantitative approach. eBioMedicine.

[37] Lui J.C. (2024). Gut microbiota in regulation of childhood bone growth. Exp. Physiol..

[38] Dash S., Syed Y.A., Khan M.R. (2022). Understanding the Role of the Gut Microbiome in Brain Development and Its Association with Neurodevelopmental Psychiatric Disorders. Front. Cell Dev. Biol..

[39] Iliodromiti Z., Triantafyllou A.R., Tsaousi M., Pouliakis A., Petropoulou C., Sokou R., Volaki P., Boutsikou T., Iacovidou N. (2023). Gut Microbiome and Neurodevelopmental Disorders: A Link Yet to Be Disclosed. Microorganisms.

[40] Warner B.B. (2019). The contribution of the gut microbiome to neurodevelopment and neuropsychiatric disorders. Pediatr. Res..

[41] Damiani F., Cornuti S., Tognini P. (2023). The gut-brain connection: Exploring the influence of the gut microbiota on neuroplasticity and neurodevelopmental disorders. Neuropharmacology.

[42] Wang Q., Yang Q., Liu X. (2023). The microbiota-gut-brain axis and neurodevelopmental disorders. Protein Cell.

[43] Liu L., Huh J.R., Shah K. (2022). Microbiota and the gut-brain-axis: Implications for new therapeutic design in the CNS. eBioMedicine.

[44] Yuan C., He Y., Xie K., Feng L., Gao S., Cai L. (2023). Review of microbiota gut brain axis and innate immunity in inflammatory and infective diseases. Front. Cell. Infect. Microbiol..

[45] Lacorte E., Gervasi G., Bacigalupo I., Vanacore N., Raucci U., Parisi P. (2019). A Systematic Review of the Microbiome in Children with Neurodevelopmental Disorders. Front. Neurol..

[46] Tognini P. (2017). Gut Microbiota: A Potential Regulator of Neurodevelopment. Front. Cell. Neurosci..

[47] Laue H.E., Coker M.O., Madan J.C. (2022). The Developing Microbiome From Birth to 3 Years: The Gut-Brain Axis and Neurodevelopmental Outcomes. Front. Pediatr..

[48] Teleanu D.M., Niculescu A.-G., Lungu I.I., Radu C.I., Vladâcenco O., Roza E., Costăchescu B., Grumezescu A.M., Teleanu R.I. (2022). An Overview of Oxidative Stress, Neuroinflammation, and Neurodegenerative Diseases. Int. J. Mol. Sci..

[49] Vacca R.A., Augello A., Gallo L., Caggianese G., Malizia V., La Grutta S., Murero M., Valenti D., Tullo A., Balech B. (2023). Serious Games in the new era of digital-health interventions: A narrative review of their therapeutic applications to manage neurobehavior in neurodevelopmental disorders. Neurosci. Biobehav. Rev..

[50] Srivastava S., Sahin M., Prock L., Nomikos G.G., Feltner D.E. (2019). Chapter 22—Translational Medicine Strategies in Drug Development for Neurodevelopmental Disorders. Handbook of Behavioral Neuroscience.

[51] Lord C., Brugha T.S., Charman T., Cusack J., Dumas G., Frazier T., Jones E.J.H., Jones R.M., Pickles A., State M.W. (2020). Autism spectrum disorder. Nat. Rev. Dis. Primers.

[52] Shaw K.A., Williams S., Patrick M.E., Valencia-Prado M., Durkin M.S., Howerton E.M., Ladd-Acosta C.M., Pas E.T., Bakian A.V., Bartholomew P. (2025). Prevalence and Early Identification of Autism Spectrum Disorder Among Children Aged 4 and 8 Years—Autism and Developmental Disabilities Monitoring Network, 16 Sites, United States, 2022.

[53] Campisi L., Imran N., Nazeer A., Skokauskas N., Azeem M.W. (2018). Autism spectrum disorder. Br. Med. Bull..

[54] Al-Beltagi M. (2021). Autism medical comorbidities. World J. Clin. Pediatr..

[55] CDC Centers for Disease Control and Prevention-Data and Statistics on ADHD. https://www.cdc.gov/adhd/data/index.html.

[56] Song P., Zha M., Yang Q., Zhang Y., Li X., Rudan I. (2021). The prevalence of adult attention-deficit hyperactivity disorder: A global systematic review and meta-analysis. J. Glob. Health.

[57] Ayano G., Demelash S., Gizachew Y., Tsegay L., Alati R. (2023). The global prevalence of attention deficit hyperactivity disorder in children and adolescents: An umbrella review of meta-analyses. J. Affect. Disord..

[58] Gnanavel S., Sharma P., Kaushal P., Hussain S. (2019). Attention deficit hyperactivity disorder and comorbidity: A review of literature. World J. Clin. Cases.

[59] Jones K.S., Saylam E., Ramphul K. (2025). Tourette Syndrome and Other Tic Disorders. StatPearls [Internet], Updated 2023 May 8 ed..

[60] Johnson K.A., Worbe Y., Foote K.D., Butson C.R., Gunduz A., Okun M.S. (2023). Tourette syndrome: Clinical features, pathophysiology, and treatment. Lancet Neurol..

[61] Ueda K., Black K.J. (2021). Recent progress on Tourette syndrome. Fac. Rev..

[62] Szejko N., Müller-Vahl K.R. (2021). Challenges in the Diagnosis and Assessment in Patients with Tourette Syndrome and Comorbid Obsessive-Compulsive Disorder. Neuropsychiatr. Dis. Treat..

[63] Apte A., Kapoor M., Naik S., Lubree H., Khamkar P., Singh D., Agarwal D., Roy S., Kawade A., Juvekar S. (2021). Efficacy of transdermal delivery of liposomal micronutrients through body oil massage on neurodevelopmental and micronutrient deficiency status in infants: Results of a randomized placebo-controlled clinical trial. BMC Nutr..

[64] Antonarakis S.E., Skotko B.G., Rafii M.S., Strydom A., Pape S.E., Bianchi D.W., Sherman S.L., Reeves R.H. (2020). Down syndrome. Nat. Rev. Dis. Primers.

[65] Onnivello S., Pulina F., Locatelli C., Marcolin C., Ramacieri G., Antonaros F., Vione B., Caracausi M., Lanfranchi S. (2022). Cognitive profiles in children and adolescents with Down syndrome. Sci. Rep..

[66] Li Q., Han Y., Dy A.B.C., Hagerman R.J. (2017). The Gut Microbiota and Autism Spectrum Disorders. Front. Cell. Neurosci..

[67] Taniya M.A., Chung H.J., Al Mamun A., Alam S., Aziz M.A., Emon N.U., Islam M.M., Hong S.S., Podder B.R., Ara Mimi A. (2022). Role of Gut Microbiome in Autism Spectrum Disorder and Its Therapeutic Regulation. Front. Cell. Infect. Microbiol..

[68] Mehra A., Arora G., Sahni G., Kaur M., Singh H., Singh B., Kaur S. (2023). Gut microbiota and Autism Spectrum Disorder: From pathogenesis to potential therapeutic perspectives. J. Tradit. Complement. Med..

[69] Chang X., Zhang Y., Chen X., Li S., Mei H., Xiao H., Ma X., Liu Z., Li R. (2024). Gut microbiome and serum amino acid metabolome alterations in autism spectrum disorder. Sci. Rep..

[70] Allan N.P., Yamamoto B.Y., Kunihiro B.P., Nunokawa C.K.L., Rubas N.C., Wells R.K., Umeda L., Phankitnirundorn K., Torres A., Peres R. (2024). Ketogenic Diet Induced Shifts in the Gut Microbiome Associate with Changes to Inflammatory Cytokines and Brain-Related miRNAs in Children with Autism Spectrum Disorder. Nutrients.

[71] Wang Y., Li N., Yang J.-J., Zhao D.-M., Chen B., Zhang G.-Q., Chen S., Cao R.-F., Yu H., Zhao C.-Y. (2020). Probiotics and fructo-oligosaccharide intervention modulate the microbiota-gut brain axis to improve autism spectrum reducing also the hyper-serotonergic state and the dopamine metabolism disorder. Pharmacol. Res..

[72] Kong X.-J., Liu J., Liu K., Koh M., Sherman H., Liu S., Tian R., Sukijthamapan P., Wang J., Fong M. (2021). Probiotic and Oxytocin Combination Therapy in Patients with Autism Spectrum Disorder: A Randomized, Double-Blinded, Placebo-Controlled Pilot Trial. Nutrients.

[73] Sanctuary M.R., Kain J.N., Chen S.Y., Kalanetra K., Lemay D.G., Rose D.R., Yang H.T., Tancredi D.J., German J.B., Slupsky C.M. (2019). Pilot study of probiotic/colostrum supplementation on gut function in children with autism and gastrointestinal symptoms. PLoS ONE.

[74] Hrnciarova J., Kubelkova K., Bostik V., Rychlik I., Karasova D., Babak V., Datkova M., Simackova K., Macela A. (2024). Modulation of Gut Microbiome and Autism Symptoms of ASD Children Supplemented with Biological Response Modifier: A Randomized, Double-Blinded, Placebo-Controlled Pilot Study. Nutrients.

[75] Wang L.J., Yang C.Y., Chou W.J., Lee M.J., Chou M.C., Kuo H.C., Yeh Y.M., Lee S.Y., Huang L.H., Li S.C. (2020). Gut microbiota and dietary patterns in children with attention-deficit/hyperactivity disorder. Eur. Child Adolesc. Psychiatry.

[76] Drechsler R., Brem S., Brandeis D., Grunblatt E., Berger G., Walitza S. (2020). ADHD: Current Concepts and Treatments in Children and Adolescents. Neuropediatrics.

[77] Cickovski T., Mathee K., Aguirre G., Tatke G., Hermida A., Narasimhan G., Stollstorff M. (2023). Attention Deficit Hyperactivity Disorder (ADHD) and the gut microbiome: An ecological perspective. PLoS ONE.

[78] Miri S., Yeo J., Abubaker S., Hammami R. (2023). Neuromicrobiology, an emerging neurometabolic facet of the gut microbiome?. Front. Microbiol..

[79] Chen Y., Xu J., Chen Y. (2021). Regulation of Neurotransmitters by the Gut Microbiota and Effects on Cognition in Neurological Disorders. Nutrients.

[80] Ast H.K., Hammer M., Zhang S., Bruton A., Hatsu I.E., Leung B., McClure R., Srikanth P., Farris Y., Norby-Adams L. (2025). Gut microbiome changes with micronutrient supplementation in children with attention-deficit/hyperactivity disorder: The MADDY study. Gut Microbes.

[81] Stevens A.J., Purcell R.V., Darling K.A., Eggleston M.J.F., Kennedy M.A., Rucklidge J.J. (2019). Human gut microbiome changes during a 10 week Randomised Control Trial for micronutrient supplementation in children with attention deficit hyperactivity disorder. Sci. Rep..

[82] Wang L.-J., Tsai C.-S., Chou W.-J., Kuo H.-C., Huang Y.-H., Lee S.-Y., Dai H.-Y., Yang C.-Y., Li C.-J., Yeh Y.-T. (2024). Add-On Bifidobacterium Bifidum Supplement in Children with Attention-Deficit/Hyperactivity Disorder: A 12-Week Randomized Double-Blind Placebo-Controlled Clinical Trial. Nutrients.

[83] Cavanna A.E., Termine C., Ahmad S.I. (2012). Tourette Syndrome. Neurodegenerative Diseases.

[84] Gill C.E., Kompoliti K. (2019). Clinical Features of Tourette Syndrome. J. Child Neurol..

[85] Geng J., Liu C., Xu J., Wang X., Li X. (2023). Potential relationship between Tourette syndrome and gut microbiome. J. De Pediatr..

[86] Wu C.-C., Wong L.-C., Hsu C.-J., Yang C.-W., Tsai Y.-C., Cheng F.-S., Hu H.-Y., Lee W.-T. (2021). Randomized Controlled Trial of Probiotic PS128 in Children with Tourette Syndrome. Nutrients.

[87] Liang Y., Wan L., Wang G., Yan H., Zhang J., Liu X., Zhang Z., Zhu G., Yang G. (2025). Clinical Study of *Limosilactobacillus reuteri* for the Treatment of Children with Chronic Tic Disorders/Tourette Syndrome: A Mid-Term Efficacy Evaluation. Neurol. Ther..

[88] Bull Marilyn J. (2020). Down Syndrome. N. Engl. J. Med..

[89] Ciciora S.L., Manickam K., Saps M. (2023). Disorders of Gut-Brain Interaction in a National Cohort of Children with Down Syndrome. J. Neurogastroenterol. Motil..

[90] Ren S., Wang X., Qin J., Mu Q., Ye S., Zhang Y., Yu W., Guo J. (2022). Altered gut microbiota correlates with cognitive impairment in Chinese children with Down’s syndrome. Eur. Child Adolesc. Psychiatry.

[91] Fisher R.S., Acevedo C., Arzimanoglou A., Bogacz A., Cross J.H., Elger C.E., Engel J., Forsgren L., French J.A., Glynn M. (2014). ILAE Official Report: A practical clinical definition of epilepsy. Epilepsia.

[92] Adejoro I.A., Babatunde D.D., Folashade O.Z., Olayiwola H.A., Johnson C.A.G., Ikechukwu A.D. (2024). Activities of Analogues of Carbamazepine and (R)–Lacosamide as Potential Inhibitors of Epilepsy Disease: Molecular Docking, DFT, and Pharmacokinetic Study. Lett. Appl. NanoBioSci..

[93] Chen Z., Brodie M.J., Liew D., Kwan P. (2018). Treatment outcomes in patients with newly diagnosed epilepsy treated with established and new antiepileptic drugs: A 30-year longitudinal cohort study. JAMA Neurol..

[94] Thuraisingam S., Salim N., Azmi I.D.M., Kassim N.K., Basri H. (2023). Development of nanoemulsion containing Centella asiatica crude extract as a promising drug delivery system for epilepsy treatment. Biointerface Res. Appl. Chem.

[95] De Caro C., Iannone L.F., Citraro R., Striano P., De Sarro G., Constanti A., Cryan J.F., Russo E. (2019). Can we ‘seize’ the gut microbiota to treat epilepsy?. Neurosci. Biobehav. Rev..

[96] Russo E. (2022). The gut microbiota as a biomarker in epilepsy. Neurobiol. Dis..

[97] Gong X., Liu L., Li X., Xiong J., Xu J., Mao D., Liu L. (2022). Neuroprotection of cannabidiol in epileptic rats: Gut microbiome and metabolome sequencing. Front. Nutr..

[98] Patel I., Mishra A., Mishra N., Kumar A. (2025). Chapter 9—Role of microbiota-gut-brain axis in epilepsy and possible interventions. Microbiota-Gut-Brain Axis and CNS Disorders.

[99] Mejía-Granados D.M., Villasana-Salazar B., Lozano-García L., Cavalheiro E.A., Striano P. (2021). Gut-microbiota-directed strategies to treat epilepsy: Clinical and experimental evidence. Seizure.

[100] Festi D., Schiumerini R., Eusebi L.H., Marasco G., Taddia M., Colecchia A. (2014). Gut microbiota and metabolic syndrome. World J. Gastroenterol..

[101] Accordino R.E., Kidd C., Politte L.C., Henry C.A., McDougle C.J. (2016). Psychopharmacological interventions in autism spectrum disorder. Expert Opin. Pharmacother..

[102] Genovese A., Butler M.G. (2020). Clinical Assessment, Genetics, and Treatment Approaches in Autism Spectrum Disorder (ASD). Int. J. Mol. Sci..

[103] Aishworiya R., Valica T., Hagerman R., Restrepo B. (2022). An Update on Psychopharmacological Treatment of Autism Spectrum Disorder. Neurotherapeutics.

[104] Caye A., Swanson J.M., Coghill D., Rohde L.A. (2019). Treatment strategies for ADHD: An evidence-based guide to select optimal treatment. Mol. Psychiatry.

[105] Ayichew T., Belete A., Alebachew T., Tsehaye H., Berhanu H., Minwuyelet A. (2017). Bacterial Probiotics their Importances and Limitations: A Review. J. Nutr. Health Sci..

[106] Mekuye B., Abera B. (2023). Nanomaterials: An overview of synthesis, classification, characterization, and applications. Nano Sel..

[107] Joudeh N., Linke D. (2022). Nanoparticle classification, physicochemical properties, characterization, and applications: A comprehensive review for biologists. J. Nanobiotechnol..

[108] Liu P., Chen G., Zhang J. (2022). A Review of Liposomes as a Drug Delivery System: Current Status of Approved Products, Regulatory Environments, and Future Perspectives. Molecules.

[109] Olusanya T.O.B., Haj Ahmad R.R., Ibegbu D.M., Smith J.R., Elkordy A.A. (2018). Liposomal Drug Delivery Systems and Anticancer Drugs. Molecules.

[110] Subramanian P. (2021). Lipid-Based Nanocarrier System for the Effective Delivery of Nutraceuticals. Molecules.

[111] da Silva Gomes A., Reis F.M.P., Ceravolo I.P., Dias-Souza M.V. (2023). Effectiveness of Free and Liposome-Entrapped Antitumoral Drugs Against Hepatocellular Carcinoma: A Comparative In Vitro Study. Biointerface Res. Appl. Chem..

[112] Yadav D., Sandeep K., Pandey D., Dutta R.K. (2017). Liposomes for Drug Delivery. J. Biotechnol. Biomater..

[113] Chime A.O., Ikechukwu V. (2013). Lipid-based drug delivery systems (LDDS): Recent advances and applications of lipids in drug delivery. Afr. J. Pharm. Pharmacol..

[114] Shade C.W. (2016). Liposomes as Advanced Delivery Systems for Nutraceuticals. Integr. Med..

[115] Najm A., Moldoveanu E.-T., Niculescu A.-G., Grumezescu A.M., Beuran M., Gaspar B.S. (2024). Advancements in Drug Delivery Systems for the Treatment of Sarcopenia: An Updated Overview. Int. J. Mol. Sci..

[116] Najm A., Bîrcă A.C., Niculescu A.-G., Alberts A., Grumezescu A.M., Gălățeanu B., Vasile B.Ș., Beuran M., Gaspar B.S., Hudiță A. (2025). Dipalmitoylphosphatidylcholine Lipid Vesicles for Delivering HMB, NMN, and L-Leucine in Sarcopenia Therapy. Molecules.

[117] Nsairat H., Khater D., Sayed U., Odeh F., Al Bawab A., Alshaer W. (2022). Liposomes: Structure, composition, types, and clinical applications. Heliyon.

[118] Tutunji L.F. (2022). Liposomes as Drug Delivery Systems. EC Pharmacol. Toxicol..

[119] Gupta A., Srivastava A., Singh A. (2020). Liposomal Drug Delivery System—A Review. J. Appl. Pharm. Sci. Res..

[120] Pande S. (2023). Liposomes for drug delivery: Review of vesicular composition, factors affecting drug release and drug loading in liposomes. Artif. Cells Nanomed. Biotechnol..

[121] Mehta M., Bui T.A., Yang X., Aksoy Y., Goldys E.M., Deng W. (2023). Lipid-Based Nanoparticles for Drug/Gene Delivery: An Overview of the Production Techniques and Difficulties Encountered in Their Industrial Development. ACS Mater. Au.

[122] Plaza-Oliver M., Santander-Ortega M.J., Lozano M.V. (2021). Current approaches in lipid-based nanocarriers for oral drug delivery. Drug Deliv. Transl. Res..

[123] Yan S., Cheng Y., Li L., Zhong C., Chen C., Gao X. (2023). Lipid-based formulations: A promising approach for poorly soluble drug delivery via the intestinal lymphatic system. J. Drug Deliv. Sci. Technol..

[124] Hashmi M.P., Koester T.M. (2018). Applications of Synthetically Produced Materials in Clinical Medicine. Reference Module in Materials Science and Materials Engineering.

[125] Gbian D.L., Omri A. (2022). Lipid-Based Drug Delivery Systems for Diseases Managements. Biomedicines.

[126] Gandek T.B., van der Koog L., Nagelkerke A. (2023). A Comparison of Cellular Uptake Mechanisms, Delivery Efficacy, and Intracellular Fate between Liposomes and Extracellular Vesicles. Adv. Healthc. Mater..

[127] Gatto M.S., Johnson M.P., Najahi-Missaoui W. (2024). Targeted Liposomal Drug Delivery: Overview of the Current Applications and Challenges. Life.

[128] Guimarães D., Cavaco-Paulo A., Nogueira E. (2021). Design of liposomes as drug delivery system for therapeutic applications. Int. J. Pharm..

[129] Duan Y., Dhar A., Patel C., Khimani M., Neogi S., Sharma P., Siva Kumar N., Vekariya R.L. (2020). A brief review on solid lipid nanoparticles: Part and parcel of contemporary drug delivery systems. RSC Adv..

[130] Mishra V., Bansal K.K., Verma A., Yadav N., Thakur S., Sudhakar K., Rosenholm J.M. (2018). Solid Lipid Nanoparticles: Emerging Colloidal Nano Drug Delivery Systems. Pharmaceutics.

[131] Satapathy M.K., Yen T.-L., Jan J.-S., Tang R.-D., Wang J.-Y., Taliyan R., Yang C.-H. (2021). Solid Lipid Nanoparticles (SLNs): An Advanced Drug Delivery System Targeting Brain through BBB. Pharmaceutics.

[132] Chaturvedi P., Sharma P. (2024). Formulation and In Vitro Evaluation of *Holoptelea integrifolia* Planch. Extract Loaded Solid Lipid Nanoparticles. Lett. Appl. NanoBioScience.

[133] Subroto E., Andoyo R., Indiarto R. (2023). Solid Lipid Nanoparticles: Review of the Current Research on Encapsulation and Delivery Systems for Active and Antioxidant Compounds. Antioxidants.

[134] Ghasemiyeh P., Mohammadi-Samani S. (2018). Solid lipid nanoparticles and nanostructured lipid carriers as novel drug delivery systems: Applications, advantages and disadvantages. Res. Pharm. Sci..

[135] Hernadez-Esquivel R.-A., Zarate-Hernández E., Navarro-Tovar G., Aguirre-Bañuelos P., Sharma A. (2022). Solid Lipid Nanoparticles (SLN). Nanocomposite Materials for Biomedical and Energy Storage Applications.

[136] Akbari J., Majid S., Fatemeh A., Hassan H.S.M., Amirhossein B., Sadra Y., Sohrab R.S., Kofi A.-A., Nokhodchi A. (2022). Solid lipid nanoparticles and nanostructured lipid carriers: A review of the methods of manufacture and routes of administration. Pharm. Dev. Technol..

[137] Cacciatore I., Michele C., Erika F., Lisa M., Di Stefano A. (2016). Solid lipid nanoparticles as a drug delivery system for the treatment of neurodegenerative diseases. Expert Opin. Drug Deliv..

[138] Mirchandani Y., Patravale V.B., Brijesh S. (2021). Solid lipid nanoparticles for hydrophilic drugs. J. Control. Release.

[139] Scioli Montoto S., Muraca G., Ruiz M.E. (2020). Solid Lipid Nanoparticles for Drug Delivery: Pharmacological and Biopharmaceutical Aspects. Front. Mol. Biosci..

[140] Al Fayez N., Böttger R., Ghosh S., Nakajima Y., Chao P.-H., Rouhollahi E., Nguyen A., Cullis P.R., Witzigmann D., Li S.-D. (2022). Development of a child-friendly oral drug formulation using liposomal multilamellar vesicle technology. Int. J. Pharm..

[141] Tang W.-L., Tang W.-H., Chen W.C., Diako C., Ross C.F., Li S.-D. (2017). Development of a Rapidly Dissolvable Oral Pediatric Formulation for Mefloquine Using Liposomes. Mol. Pharm..

[142] Chandramouli M., Basavanna V., Ningaiah S. (2024). A Comprehensive Review of Paediatric Drug Development: An Extensive Analysis of Present Difficulties and Prospects for the Future. Biointerface Res. Appl. Chem..

[143] Zhang Y. Phase II Randomized Controlled Clinical Trial of Mitoxantrone Hydrochloride Liposome Combined with Irinotecan and Vincristine (VIM) with VIT in Children with Relapsed and Refractory Soft Tissue Sarcoma. https://clinicaltrials.gov/study/NCT06514313?cond=Children&term=Liposome&rank=3.

[144] St. Jude Children’s Research Hospital A Protocol for the Treatment of Newly Diagnosed Rhabdomyosarcoma Using Molecular Risk Stratification and Liposomal Irinotecan Based Therapy in Children with Intermediate and High Risk Disease. https://clinicaltrials.gov/study/NCT06023641?cond=Children&term=Liposome&rank=9.

[145] Namita Mishra, All India Institute of Medical Sciences Effect of Ferrous Ascorbate Versus Liposomal Iron on Hemoglobin Concentration and Iron Indices in 6 to 59 Months Age Children with Nutritional Iron-Deficiency Anemia: A Double-Blinded Single Centre Randomized Clinical Trial. https://clinicaltrials.gov/study/NCT05957328?cond=Children&term=Liposome&rank=10.

[146] Alexandra Stevens, Baylor College of Medicine A Trial of Atovaquone (Mepron®) Combined with Conventional Chemotherapy for De Novo Acute Myeloid Leukemia (AML) in Children, Adolescents, and Young Adults (ATACC AML). https://clinicaltrials.gov/study/NCT03568994?cond=Children&term=Liposome&page=3&rank=22.

[147] University of Birmingham International Randomised Phase III Clinical Trial in Children with Acute Myeloid Leukaemia—Incorporating an Embedded Dose Finding Study for Gemtuzumab Ozogamicin in Combination with Induction Chemotherapy. https://clinicaltrials.gov/study/NCT02724163?cond=Children&term=Liposome&page=3&rank=30.

[148] Cao Z., Wang X., Pang Y., Cheng S., Liu J. (2019). Biointerfacial self-assembly generates lipid membrane coated bacteria for enhanced oral delivery and treatment. Nat. Commun..

[149] Chowdhuri S., Cole C.M., Devaraj N.K. (2016). Encapsulation of Living Cells Within Giant Phospholipid Liposomes Formed by the Inverse-Emulsion Technique. ChemBioChem.

[150] Azeem M., Farhan S., Muhammad A., Huda A., Aftab A., Atif L., Rosa B., Lorenzo J.M., Asif Shah M. (2023). Encapsulation of probiotics in solid lipid micro particle for improved viability and stability under stressed conditions. Int. J. Food Prop..

[151] Kumar N., Tyagi N., Mehan S., Singh A., Verma B., Kumar S. (2024). Preparation of probiotic-loaded solid lipid nanoparticles and in vitro survival in gastrointestinal conditions. BIO Web Conf..

[152] Han M., Yang S., Song J., Gao Z. (2024). Layer-by-layer coated probiotics with chitosan and liposomes demonstrate improved stability and antioxidant properties in vitro. Int. J. Biol. Macromol..

